# Spectrum of genetic variants in moderate to severe sporadic hearing loss in Pakistan

**DOI:** 10.1038/s41598-020-68779-5

**Published:** 2020-07-17

**Authors:** Memoona Ramzan, Rasheeda Bashir, Midhat Salman, Ghulam Mujtaba, Nara Sobreira, P. Dane Witmer, Baylor-Hopkins Center for Mendelian Genomics, Sadaf Naz

**Affiliations:** 1 School of Biological Sciences, University of the Punjab, Quaid-i-Azam campus, Lahore, 54590 Pakistan; 2 McKusick-Nathans Department of Genetic Medicine, Johns Hopkins University, Baltimore, MD USA; 3 Johns Hopkins Genomics, Johns Hopkins University, Baltimore, MD USA; 4 Present Address: Department of Biotechnology, Lahore College for Women University, Lahore, Pakistan; 5 Present Address: Virtual University of Pakistan, Lahore, Pakistan; 6 Present Address: Institute of Nuclear Medicine and Oncology (INMOL), Lahore, Pakistan

**Keywords:** Neuroscience, Auditory system, Consanguinity, Genetic testing, Genetics, Sequencing, DNA sequencing, Next-generation sequencing

## Abstract

Hearing loss affects 380 million people worldwide due to environmental or genetic causes. Determining the cause of deafness in individuals without previous family history of hearing loss is challenging and has been relatively unexplored in Pakistan. We investigated the spectrum of genetic variants in hearing loss in a cohort of singleton affected individuals born to consanguineous parents. Twenty-one individuals with moderate to severe hearing loss were recruited. We performed whole-exome sequencing on DNA samples from the participants, which identified seventeen variants in ten known deafness genes and one novel candidate gene. All identified variants were homozygous except for two. Eleven of the variants were novel, including one multi-exonic homozygous deletion in *OTOA*. A missense variant in *ESRRB* was implicated for recessively inherited moderate to severe hearing loss. Two individuals were heterozygous for variants in *MYO7A* and *CHD7*, respectively, consistent with de novo variants or dominant inheritance with incomplete penetrance as the reason for their hearing loss. Our results indicate that similar to familial cases of deafness, variants in a large number of genes are responsible for moderate to severe hearing loss in sporadic individuals born to consanguineous couples.

## Introduction

Approximately 50% of hearing loss cases has a genetic etiology. Of these, 70% are nonsyndromic and the remaining 30% are syndromic^1,2^. Nonsyndromic hearing loss is most frequently inherited as an autosomal recessive trait^3^ and the genetics of profound deafness in families has been well characterized^4^. However, few studies have explored the prevalence of sporadic, moderate to severe hearing loss in the broader population.

De novo variants or variants with incomplete penetrance, recessively inherited X-linked variants and mitochondrial variants may occur in individuals with no family history of hearing loss^5,6^. The etiology of severe to profound deafness in sporadic cases has been explored in some populations and variants in *GJB2* (OMIM 121011) and *SCL26A4* (OMIM 605646) have been found to be the major contributors^6–9^. Worldwide, only a few comprehensive studies have been carried out on single affected individuals exhibiting moderate to severe hearing loss. These studies have shown that variants in *GJB2*, *OTOG* (OMIM 604487), *STRC* (OMIM 606440), *TECTA* (OMIM 602574) and *SERPINB6* (OMIM 173321) are more frequent in individuals with moderate or moderate to severe hearing loss in Chinese, Korean, Japanese and American populations^5,10–12^. So far, no extensive study has been conducted to elucidate the genetic etiology of moderate to severe, sporadic hearing loss in Pakistan. We present the first report on the contribution of different deafness genes in the etiology of hearing loss of sporadic individuals born to consanguineous parents.

## Methods

### Ascertainment and audiological assessment

This study was approved by Institutional Review Board of School of Biological Sciences (SBS), University of the Punjab, Lahore, Pakistan. All the experiments were performed in accordance to the guidelines and regulations set by the relevant body. Twenty-one probands were identified from various schools for deaf and special education schools in Punjab. The selected participants were born to consanguineous parents. The syndromic associations with hearing loss in this cohort were excluded by observation of the clinical features in individuals and detailed questioning regarding phenotypes. Written informed consents were obtained from participants or their legal guardians.

Audiometry was performed to measure average hearing thresholds for all the participants at frequencies of 0.5, 1, 2, 4 and 8 kHz under ambient noise conditions. Romberg and tandem gait tests were completed to identify vestibular defects.

### Whole-exome sequencing and variants filtering

Whole blood up to 10 ml was drawn from the participants and the DNA was extracted using a standard method involving sucrose lysis and salting out. Whole-exome sequencing was performed at the Baylor-Hopkins Center for Mendelian Genomics (BHCMG). Exome capture was performed using the Agilent SureSelect Human All ExonV5 kit using a low input library preparation protocol^13^. Libraries were sequenced on the Illumina HiSeq2500 platform to generate 125 bp paired end runs. Reads were aligned with BWA mem 0.7.8 to the 1,000 genomes phase 2 (GRCh37) human genome reference sequence. Variant calling was performed using GATK 3.3–0 joint calling with HaplotypeCaller. The data after final output was analyzed independently at BHCMG, USA and SBS, University of the Punjab, Lahore. The program wANNOVAR (https://wannovar.usc.edu/) was used for annotating the variant call files (VCF). Using either the PhenoDB Variant Analysis Tool^14^ or manually, the output data from wANNOVAR was filtered against the population frequencies in the 1,000 Genomes database, genome Aggregation Database (gnomAD) and the Exome Aggregation Consortium (ExAC) database. Variants were retained for further evaluation if they had an allele frequency of less than 0.01 in these public databases. Homozygous, hemizygous and compound heterozygous exonic and splice site variants were examined. Large deletions and copy number variations (CNV) were detected using ExomeDepth^15^ using read depth data from exome sequencing experiments. For these analyses, each test exome was compared to a matched, aggregate reference set of samples. CNV calls were annotated using AnnotSV^16^. Candidate CNVs were prioritized by minor allele frequency, exon number, Bayes factor (BF) and the ratio of observed/expected number of reads.

The wANNOVAR files also included predicted pathogenic scores for these variants from Polyphen 2, Mutation Taster and SIFT along with the pathogenicity CADD scores indicating the probable impact of variation on the function of the encoded protein. In addition, REVEL pathogenicity scores for the variants were accessed (https://sites.google.com/site/revelgenomics/). The conservation of selected amino acid residues affected by variants was checked across vertebrate species. For this purpose, multiple alignments were carried out on the protein sequences obtained from UniProt (https://www.uniprot.org/) using ClustalO (https://www.ebi.ac.uk/Tool/msa/clustalo).

## Results

### Subjects and audiological phenotype

**Figure 1:**
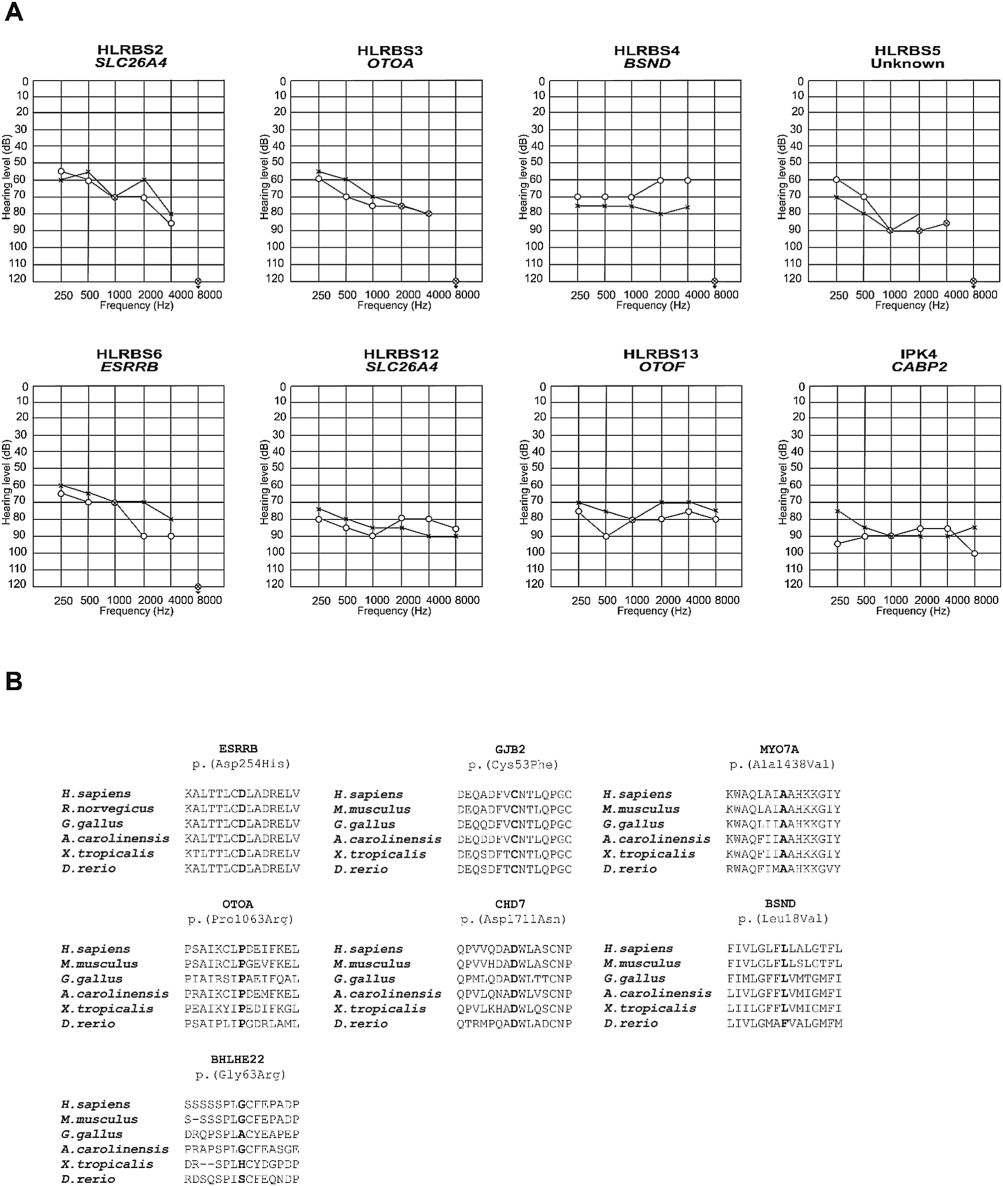
Audiograms for selected participants and partial protein sequence alignments from diverse vertebrate species. (**A**) Audiometry was performed under ambient noise conditions and hearing threshold is classified as moderate to severe or severe. (**B**) Clustal alignments show conservation of amino acids affected by novel missense variants. The residues are indicated in bold. All amino acids affected by variants are conserved except for one in *BSND* and *BHLHE22*. ‘o’ right ear, ‘x’ left ear.

Twenty-one individuals including sixteen males and five females with ages ranging from 5 to 23 years participated in the study. The pure tone averages (PTA) for better hearing ears ranged between 65 to 88 dB HL (Fig. 1). Romberg and tandem gait tests were negative, which indicated normal vestibular function. All the participants exhibited no other phenotype except for hearing loss at the time of recruitment.

### Genetic findings after whole-exome sequencing

Whole-exome sequencing identified candidate causative variants in eleven genes in seventeen individuals (Table 1). Of the seventeen identified variants, fifteen were classified as pathogenic based on the predictions from in silico tools and pathogenicity scores from REVEL and CADD. According to the guidelines by ACMG^17^ twelve were pathogenic, three were likely pathogenic and two variants were classified as variants of uncertain significance. Most of the variants except for one variant in *OTOF* (OMIM 603681) were unique to only one individual. All variants observed during this study have been deposited in ClinVar (SCV000924172.1, SCV000924173.1, SCV000924174.1, SCV000924175.1, SCV000924197.1, SCV000924198.1, SCV000924201.1, SCV000924202.1, SCV000924203.1, SCV000929840.1, SCV000929841.1, SCV000929842.1, SCV000929844.1, SCV000929845.1 and SCV000929852.1). No candidate variants were obtained from four participants.

**Table 1:** Details of genes and variants obtained from the analysis of whole-exome sequencing.

Case ID	Gene	Transcript/Variant	Variant effect	Genotype	Pathogenicity			REVEL Score	Global frequency		First report/PMID
					SIFT	PolyPhen2	MT		ExAC	gnomAD	
HLRBS13 HLRBS14	*OTOF*	NM_001287489/c.4490_4491del p.(Tyr1497TyrfsTer10)	Frameshift	Homo	N/A,N/A,D			N/A	–	–	This study
				Homo	N/A,N/A,D			N/A	–	–	This study
SPK6		c.2443delC p.(Gln815GlnfsTer1)		Homo	N/A,N/A,D			0.41	–	–	This study
SPK5	*GJB2*	NM_004004/c.158G > T p.(Cys53Phe)	Missense	Homo	D,D,D			0.95	–	–	This study
SPK1		c.370C > T p.(Gln124Ter)	Nonsense	Homo	D,N/A,D			N/A	0.00009	0.00008	9600457
HLMS29	*BSND*	NM_057176/c.52G > C p.(Leu18Val)	Missense	Homo	T,D,N			0.40	0.00006	0.00006	VUS/This study
HLRBS4		c.35 T > C p.(Ile12Thr)	Missense	Homo	D,D,D			0.81	0.00002	0.00001	19646679
HLRBS2	*SLC26A4*	NM_000441/c.1692dupA p.(Cys565PhefsTer10)	Frameshift	Homo	N/A,N/A,D			N/A	–	–	18167283
HLRBS12		c.269C > T p.(Ser90Leu)	Missense	Homo	D,D,D			0.89	0.00008	0.00001	12676893
HLRBS3	*OTOA*	NM_144672/c.3188C > G p.(Pro1063Arg)	Missense	Homo	D,D,D			0.25	–	–	This study
SPK7		NC_000016/g.(21575218_21624036)_(21747738_21777910) del	Large deletion	Homo	–			–	–	–	This study
HLMS31	*RDX*	NM_001260492/c.551 + 2 T > C	Splicing	Homo	N/A,N/A,D			N/A	–	–	This study
HLMS32	*MYO7A*	NM_000260/c.4313C > T p.(Ala1438Val)	Missense	Het	D,B,D			0.75	0.00001	0.000	VUS/This study
IPK4	*CABP2*	NM_001318496/c.637 + 1G > T p.(Phe164SerfsTer4)	Splicing	Homo	N/A,N/A,D			N/A	0.0011	0.0010	22981119
HLGMSA01	*CHD7*	NM_017780/c.5131G > A p.(Asp1711Asn)	Missense	Het	T,D,D			0.39	0.00001	0.000008	This study
HLMS7	*BHLHE22*	NM_157414/c.187G > C p.(Gly63Arg)	Missense	Homo	D,P,N			0.38	0.00008	0.0001	This study
HLRBS6	*ESRRB*	NM_004452/c.733G > C p.(Asp245His)	Missense	Homo	D,D,D			0.89	–	–	This study
IPK5	Unknown										
HLRBS5											
HLRBS21											
HLMS1											

*N/A* Not applicable, *D* disease causing/deleterious/damaging, *P* probably damaging, *N* neutral, *T* tolerated, *B* benign, *VUS* variant of uncertain significance, *REVEL* rare exome variant ensemble learner, *SIFT* sorting intolerant from tolerant, *MT* mutation taster, *PMID* PubMed identifier, *ExAC* Exome Aggregation Consortium, *gnomAD* genome aggregation database.

### Pathogenic homozygous variants in genes known to cause nonsyndromic deafness

Variants in *OTOF* affected three individuals with deafness in this cohort. Two novel frameshift variants were identified for three unrelated participants. The variant c.4990_4991del (p.Tyr1497TyrfsTer10) was present in two individuals HLRBS13 and HLRBS14. Another novel frameshift variant c.2443delC (p.Gln815GlnfsTer1) was identified in individual SPK6. Variants in *GJB2*, *SLC26A4* and *OTOA* (OMIM 607038) affected more than one individual. Among these, one missense variant c.158G > T (p.Cys53Phe) in *GJB2* and one variant c.3188C > G (p.Pro1063Arg) in *OTOA* were identified for the first time. A large homozygous deletion including *OTOA* was identified in individual SPK7. Homozygous missense variants in *RDX* (OMIM 179410), *CABP2* (OMIM 607314), and *ESRRB* (OMIM 602167) were found to contribute to hearing loss in three individuals. All these variants had high pathogenicity scores and the affected amino acids were conserved among different vertebrate species (Fig. 1B).

### Pathogenic homozygous variants in syndromic deafness gene

One known and one novel homozygous missense variant in exon 1 of *BSND* (OMIM 606412) were identified in two unrelated individuals. The characteristic symptoms of Barter’s syndrome such as polyhydramnios or salt loss during neonatal stages were ruled out in both individuals by detailed questioning from the mothers of the affected children. The variant c.35 T > C (p.Ile12Thr) has been reported to cause nonsyndromic deafness DFNB73 (OMIM 602522)^18^. Since the novel variant c.52G > C (p.Leu18Val) affects an amino acid located in close proximity of the reported p.Ile12Thr variant, it is possible that p.Leu18Val is also a nonsyndromic deafness allele. However, some individuals with missense variants only manifest renal abnormalities as adults^19^. It is therefore possible that renal abnormalities could be manifested in future by the child with the p.(Leu18Val) variant in *BSND*.

### Pathogenic heterozygous variants

A heterozygous variant c.1531G > A (p.Asp1711Asn) in *CHD7* (OMIM 608892) was identified in individual HLGMSA01. The variant was predicted to be damaging by three of the four prediction tools. No characteristic features of CHARGE syndrome (OMIM 214800) such as unusual shape of external ear, facial asymmetry, small eyes, delayed growth and delay in development of motor skills were observed for the affected individual or reported by the parents and siblings at the time of sampling. Samples from parents and siblings were not available to establish the segregation of the variant and to determine if it was a de novo variant.

A heterozygous variant c.4313C > T (p.Ala1438Val) in *MYO7A* (OMIM 276903) was identified in HLMS32. The variant had high REVEL (0.75) and CADD scores (25.8), and was predicted to be damaging by three of the mentioned prediction tools.

### Missense variant in a novel candidate gene

A missense variant, c.187G > C (p.Gly63Arg) in *BHLHE22* (OMIM 613483) was identified in individual HLMS7 who had moderate to severe hearing loss. This variant was predicted to be disease causing by various online prediction tools but was of uncertain significance according to ACMG guidelines. The variant had a high CADD score of 22.9 and a relatively low REVEL score of 0.38. The variant was rare as it had a low allele frequency of 0.00008 and 0.0001 in ExAC and gnomAD respectively, with no homozygous individuals in the control population. It had a GERP++ score of 3.18.

## Discussion

Consanguineous families have served as a rich resource for the identification of genetic causes of recessively inherited disorders. In Pakistan 40–60% marriages are among first cousins^20,21^ which increases the risk of prevalence of recessive disorders, including hearing loss. According to the World Health Organization, the Pakistani population has a high prevalence of recessive disorders (2.4%) as compared to the incidence worldwide (1.7%). In this study, we explored the genetics of moderate to severe hearing loss for the first time in Pakistan in single individuals born to unaffected parents who were cousins. It was suspected that variants in few genes like *STRC*, *GJB2*, *SLC26A4*, *OTOG* or *TECTA* may explain the hearing loss for the majority of individuals in our cohort as is the case in many other world populations for the individuals with moderate to severe deafness. However, the identification of variants in multiple genes associated with hearing loss in our cohort of sporadic cases suggests a similar genetic heterogeneity in sporadic and familial cases in Pakistan. The combined contribution of genes involved in profound deafness is 52% to moderate to severe hearing loss in this cohort. The phenotypic variability due to variants in the same genes implicate the involvement of extrinsic factors or modifiers affecting the severity of hearing loss.

The variants in *OTOF* were more frequent in our cohort as compared to the published data for different ethnicities or populations. Four other reports from Korea, Japan and China on the genetic predisposition of hearing loss in sporadic individuals included more than 60 participants with moderate to severe or profound deafness, in which they demonstrated *SLC26A4* as the major contributor to hearing loss^6,22,23^.

The *GJB2* related deafness accounts for 10% cases in our cohort, which is similar to the reported incidence of *GJB2* variants (9.5%) obtained from screening of large consanguineous families^11^. However, a recent research from Pakistan on 40 individuals with profound deafness from Bannu and Kohat districts indicated that *GJB2* variants caused deafness in 37% of non-familial cases^9^. The small sample size, difference of ethnic background and less severe hearing phenotype may explain this lower contribution of *GJB2* variants in the present study.

A variant c.733G > C (p.Asp245His) in *ESRRB* was identified for moderate to severe hearing loss in one participant of this study. *ESRRB* is an estrogen related receptor beta gene which is known to cause hearing loss at DFNB35 (OMIM 608565) locus. The encoded protein consists of two domains; DNA binding domain (DBD) and ligand binding domain (LBD). Seven of the previously identified variants affect the ligand binding domain of the protein. The variant identified in this study also affects the ligand binding domain and the amino acid at this position is conserved among vertebrate orthologues (Fig. 1B). However, instead of profound deafness, the missense variant was observed to cause a moderate to severe phenotype in the affected individual in this study. It suggests that the severity of hearing loss caused by *ESRRB* can be modified by certain genetic or environmental factors.

A missense variant c.187G > C (p.Gly63Arg) in *BHLHE22* was potentially implicated for moderate to severe hearing loss. *BHLHE22* has a single coding exon which encodes a class E basic helix loop helix protein 22 (BHLHE22). It is a small protein of 381 amino acids which serves as a sequence specific DNA binding transcription factor and mediates cell differentiation and proliferation. Mutant murine models have demonstrated that BHLHE22 is necessary for retinogenesis^24^ and development of dorsal cochlear nuclei^25,26^. *BHLHE22* has the highest expression in retina^26^ however, it is also expressed in cochlear hair cells, supporting cells and utricle in the inner ear (umgear.org, https://shield.hms.harvard.edu/).

The variant c.187G > C in *BHLHE22* had a relatively low conservation score (3.18; only conserved among mammals and some reptiles) and high pathogenicity scores (CADD, 22.9). These scores may be explained by a previous study on transcriptional repressors, which suggested that nonconserved regions are vital for the DNA binding function of the proteins. They may also provide a drift during evolution for the correct folding and thus secondary structure of the respective protein^27^. Therefore, the identification of *BHLHE22* variants in additional affected individuals or mice models will be useful to understand the role of this gene, if any, in hearing loss.

Majority of variants in *MYO7A* primarily cause autosomal recessive nonsyndromic hearing loss (DFNB2) (OMIM 600060) and Usher syndrome 1B (USH1B) (OMIM 27690)^28,29^. A heterozygous variant identified in individual HLMS32 suggests that hearing loss is probably nonsyndromic dominant as observed for DFNA11 (OMIM 601317) instead of USH1B or DFNB2 which are caused by biallelic variants of *MYO7A*. However, we cannot exclude the possibilities that either the individual is a carrier for DFNB2/USH1B variant or the variant may be benign, in spite of its prediction to be damaging.

The diagnostic rate for sporadic cases in this research was relatively high (80%) as compared to other studies. For instance, in a cohort of 63 simplex cases from China the successful diagnostic rate was of 12.7%^8^. Few other studies on sporadic cases from Korea, China and Japan have reported the pathogenic variant detection rates of 20% (from 92 cases)^30^, 23.1% (from 13 cases)^6^, 32% (from 34 cases)^31^ and 45.4% (from 11 cases)^5^ using whole-exome sequencing. The mutation detection rate in these studies is lower as they screened the individuals for common variants of *GJB2* and *SLC26A4* prior to whole-exome sequencing. However, even after excluding the *GJB2* cases, the identification rate for the present study still remains as high as 71%. This may perhaps be due to the fact that we specifically studied hearing loss in individuals born in consanguineous unions. This increased the possibility that the disorder was recessively inherited.

Variants of uncertain significance were identified in multiple participants (Table 1) while no potential pathogenic variant was identified for four individuals after whole-exome sequencing. For the latter, some pathogenic variants may have been overlooked due to the stringent criteria to classify a variant as pathogenic. Secondly, a few pathogenic variants may have been missed as they could be present in non-coding exons, introns or regulatory regions of the genes.

Our study comprehensively evaluated the genetic cause of moderate to severe hearing loss in a cohort of sporadic individuals. Results show that a similar diversity of gene variants is responsible for sporadic deafness as seen for familial hearing loss. Therefore, such cohorts can serve as a rich source for the determination of genetic and molecular basis of hereditary deafness. These results also suggest that targeted sequencing of few common deafness genes prioritized according to the ethnicity, followed by whole-exome sequencing will be a simple and cost effective approach for the genetic diagnosis and management of isolated hearing loss.
